# Characterization of *Haemaphysalis flava* (Acari: Ixodidae) from Qingling Subspecies of Giant Panda *(Ailuropoda melanoleuca qinlingensis)* in Qinling Mountains (Central China) by Morphology and Molecular Markers

**DOI:** 10.1371/journal.pone.0069793

**Published:** 2013-07-19

**Authors:** Wen-yu Cheng, Guang-hui Zhao, Yan-qing Jia, Qing-qing Bian, Shuai-zhi Du, Yan-qing Fang, Mao-zhen Qi, San-ke Yu

**Affiliations:** College of Veterinary Medicine, Northwest A&F University, Yangling, Shaanxi Province, China; Institut national de la santé et de la recherche médicale - Institut Cochin, France

## Abstract

Tick is one of important ectoparasites capable of causing direct damage to their hosts and also acts as vectors of relevant infectious agents. In the present study, the taxa of 10 ticks, collected from Qinling giant pandas (*Ailuropoda melanoleuca qinlingensis*) in Qinling Mountains of China in April 2010, were determined using morphology and molecular markers (nucleotide ITS2 rDNA and mitochondrial 16S). Microscopic observation demonstrated that the morphological features of these ticks were similar to *Haemaphysalis flava*. Compared with other *Haemaphysalis* species, genetic variations between *Haemaphysalis* collected from *A. m. qinlingensis* and *H. flava* were the lowest in ITS2 rDNA and mitochondrial 16S, with sequence differences of 2.06%–2.40% and 1.30%–4.70%, respectively. Phylogenetic relationships showed that all the *Haemaphysalis* collected from *A. m. qinlingensis* were grouped with *H. flava*, further confirmed that the *Haemaphysalis* sp. is *H. flava*. This is the first report of ticks in giant panda by combining with morphology and molecular markers. This study also provided evidence that combining morphology and molecular tools provide a valuable and efficient tool for tick identification.

## Introduction

**Figure 1:**
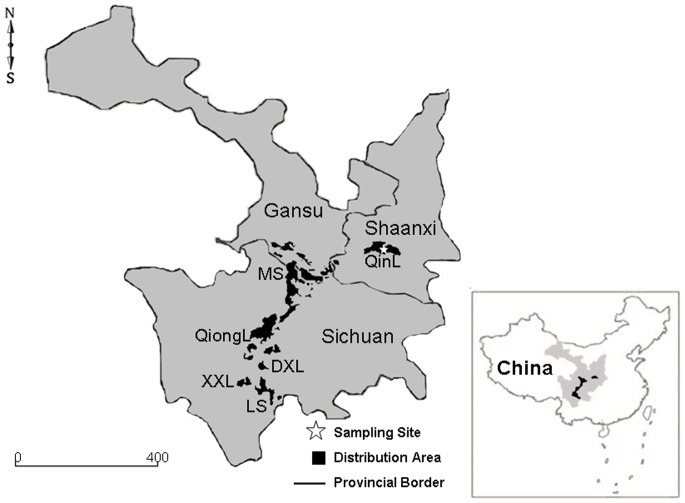
Sampling sites for ticks (asterisk) and distributions of giant pandas (black color) in China. QinL, Qinling Mountains; MS, Minshan Mountains; QiongL, Qionglai Mountains; DXL, Daxiangling Mountains; XXL, Xiaoxiangling Mountains; LS, Liangshan Mountains.

The giant panda (*Ailuropoda melanoleuca*) is one of the most endangered and rarest animals in the world [1], [2]. It was considered as Category I in the List of Key Protected Wildlife, an appendix within the Law of the People’s Republic of China on the Protection of Wildlife in 1989, and was also listed by the IUCN Species Survival Commission (SSC) as “Rare” in 1986 and 1988 and “Endangered” in 1990, 1994, and 1996 [3], [4]. The total population of giant pandas has been estimated to approximately 1600 reported in Qinling, Minshan, Qionglai, Daxiangling, Xiaoxiangling and Liangshan Mountains in China (Figure 1) [5], [6]. Since 1957, many conservation projects have been carried out, but the species is still facing the crisis of extinction because of habitat loss, poor reproduction and low rates of infectious disease resistance [4], [7], [8]. Among them, parasitic infection is the most endangered factor for giant pandas in China [9], e.g. ascariasis, some trematodiasis, diseases and infestations by ectoparasites [10].

Tick (Ixodida), one of common ectoparasites in animals, is an obligatory blood-feeding parasite capable of infesting all terrestrial vertebrates and birds, and is also globally important as vector of many microorganisms [11]. They are important for human and animal health, and can also cause high economic losses [12]. As a kind of widespread ectoparasites, the hard tick was firstly reported in giant pandas by Wu et al. in 1984 [13], latterly detailed morphological characters described and identified as *Haemaphysalis warburtoni*. These ticks could attach to the skin of giant pandas, and cause dermatitis, and, in highly infested animals weight loss, anemia and even death [10]. Ticks also can cause secondary skin bacterial infections [10]. According to a pathological examination, nearly 100 ticks have been collected in giant pandas sheltered in Pingwu Nature Reserve of Sichuan province the tick fauna consists of 5 species belonging to 2 genera, namely *Ixodes* and *Haemaphysalis* [14]. Subsequently, Qiu et al. [15] collected 1135 hard ticks from 11 wild giant pandas belonging to 12 species and 3 genera, namely, *Ixodes* (3 species), *Haemaphysalis* (8 species) and *Dermacentor* (1 species). Ticks belonging to genera *Ixodes* (3 species) and *Haemaphysalis* (8 species) were also found in dead giant pandas in Wen county of Gansu province [16]. In 1990, three hard tick species, namely *I. ovatus*, *H. aponommoides* and *H. flava* were also isolated from rescued giant pandas in Sichuan province [17]. Nowadays, more than 12 tick species have been identified as infesting giant panda based on morphological features [18].

Although morphological approach for tick identification is economic and convenient, it is expert-needing, inaccurate for close-related species, and also difficult for tick species infesting wild animals [19]. Molecular approach, mainly based on mitochondrial (mt) and ribosomal DNA (rDNA) fragments, has provided complementary tool for accurate identification and characterization of pathogens [20]–[24]. The internal transcribed spacer (ITS) rDNA, one highly tandem repeat region in genome, has been identified as one of the most effective marker for phylogenetics, diagnostics and DNA barcoding [25]. The ITS2 rDNA has been applied for studying molecular evolution of ixodidae and identifying tick species with length polymorphisms [26]–[29]. The ITS2 rDNA also has been successfully used to differentiate morphologically similar tick species of *Rhipicephalus sanguineus* group, *Ixodes* spp. and *Amblyomma* spp. [30]–[32]. Due to higher mutation rate, maternal inheritance and haploid nature, the mtDNA has been identified as a species-level marker for phylogenetic and taxonomic studies of organisms [33]–[35]. Four mtDNA fragments, namely 12S, 16S, cytochrome c oxidase subunit 1 (*cox*1) and NADH dehydrogenase subunit 5 (*nad*5), could be used to infer phylogenetic studies of ticks [36]–[40]. The mt 16S, 12S and *cox*1 were also acted as good molecular targets to identify new species of argasid ticks and *Ixodes* spp. [22], [41]. However, previous study showed that the ITS rDNA is useful to approach taxonomic and phylogenetic questions in arthropods, and the mt 16S is more suitable for solving taxonomic and phylogenetic problems between closely-related species [42]. Using ITS2 rDNA and mt 16S sequences, the phylogenetic relationships of *Amblyomma* species were effectively re-constructed [43], and four Euro-Asian hard tick species (*I. ricinus*, *I. persulcatus*, *I. hexagonus*, and *D. reticulatus*) were identified and differentiated [44].

The objective of this study was to identify ticks collected from giant pandas (*A. m. qinlingensis*) in Qinling Mountains in Shaanxi province of China, using morphological and molecular approaches. Based on ITS2 rDNA and mt 16S sequences, the phylogenetic relationships of these ticks were also re-constructed.

## Materials and Methods

### Ethics Statement

The performance of this study was strictly according to the recommendations of the Guide for the Care and Use of Laboratory Animals of the Ministry of Health, China, and our protocol was reviewed and approved by the Research Ethics Committee of Northwest A&F University. All tick samples were collected from *A. m. qinlingensis* after the permission of the Shaanxi Rare Wildlife Rescue Breeding Research Center, with no specific permits being required by the authority for the tick collection.

### Tick Collection

In April 2010, a total of 30 adult hard ticks were collected from four *A. m. qinlingensis* (2 males and 2 females, 8–12 years old) using forceps without damaging their mouthparts. These giant pandas were rescued from Qinling Mountains in Shaanxi province of China (Figure 1). Collected specimens were conserved in 70% ethanol and taken to the laboratory for further examination.

### Morphological Identification

Ticks were identified based on existing keys and descriptions [45]. Briefly, measurements for adults (males and females) were made using a stereomicroscope and provided in millimeters. Two specimens of each sex were put on glass slides and examined by light microscopy (OLYMPUS-CH20 BIM, 40×) for morphological analyses and morphometry.

### Molecular Identification

Considering similar morphological features of these ticks, 10 adult ticks (3 females and 7 males) were selected for molecular analysis (Table 1). The genomic DNA was individually extracted from ticks using spin column purification (TIANamp Genomic DNA Purification System, TIANGEN, China) according to the manufacturer’s recommendations. Molecular characterizations of ticks were performed by sequences of ITS2 rDNA and mt 16S. PCR amplification of the ITS2 rDNA was conducted according to Chitimia et al. (2009) by using primers TITS2F1 (5′-CGAGACTTGGTGTGAATTGCA-3′) and TITS2R1 (5′-TCCCATACACCACATTTCCCG-3′) [46]. The oligonucleotide primer set of mt 16S, namely 16Su (5′-GTAGGATTTCAAAAGTTGAACAAACTT-3′) and 16Sd (5′-CAATGAATATTTAAATTGCTGTAGT-3′), were designed based on available mt 16S sequences of *Haemaphysalis* spp. in GenBank™. Polymerase chain reactions were performed in 25 µl reaction system containing 1×Taq MasterMix (CWBIO, China), 0.4 mM of each primer with 2 µl of gDNA in a thermocycler (Eppendorf) under the following conditions: denaturation at 95°C for 5 min, followed by 40 cycles of 94°C for 45 s, 55°C for 1 min, 72°C for 90 s, and a final extension at 72°C for 5 min. Samples without gDNA and host DNA (*A. m. qinlingensis*) as negative controls were also included in each amplification. Each amplicon (5 µl) was examined by agarose gel (1%) electrophoresis to validate amplification efficiency. Positive amplicons were sent to Sangon Company (Shanghai, China) for sequencing.

**Table 1:** Information of tick samples used in the present study.

Species/Sample codes	Gender	Host	Geographical origin		GenBank™ accession number	
			ITS-2	16S	ITS-2	16S
*Dermacentor andersoni*	–	–	Germany	Canada (British Columbia)	S83084	EU711328
*Haemaphysalis doenitzi*	–	–	China (Sichuan)	China (Sichuan)	JQ346685	JF979402
*H. elliptica*	–	–	–	South Africa	–	HM068961
*H. flava*	–	–	China (Hubei)	Japan (Saitama)	JF758641	NC_005292
*H. flava*	–	–	China (Hubei)	–	JQ625711	–
*H. flava*	–	–	China (Hubei)	–	JQ625712	–
*H. flava*/HFS1	Female	Giant panda	China (Shaanxi)	China (Shaanxi)	**KC844868**	**KC844858**
*H. flava*/HMS1	Male	Giant panda	China (Shaanxi)	China (Shaanxi)	**KC844869**	**KC844859**
*H. flava*/HFS2	Female	Giant panda	China (Shaanxi)	China (Shaanxi)	**KC844870**	**KC844860**
*H. flava*/HMS2	Male	Giant panda	China (Shaanxi)	China (Shaanxi)	**KC844871**	**KC844861**
*H. flava*/HFS3	Female	Giant panda	China (Shaanxi)	China (Shaanxi)	**KC844872**	**KC844862**
*H. flava*/HMS3	Male	Giant panda	China (Shaanxi)	China (Shaanxi)	**KC844873**	**KC844863**
*H. flava*/HMS4	Male	Giant panda	China (Shaanxi)	China (Shaanxi)	**KC844874**	**KC844864**
*H. flava*/HMS5	Male	Giant panda	China (Shaanxi)	China (Shaanxi)	**KC844875**	**KC844865**
*H. flava*/HMS6	Male	Giant panda	China (Shaanxi)	China (Shaanxi)	**KC844876**	**KC844866**
*H. flava*/HMS7	Male	Giant panda	China (Shaanxi)	China (Shaanxi)	**KC844877**	**KC844867**
*H. humerosa*	–	–	–	–	AF199115	JX573138
*H. hystricis*	–	–	–	–	–	JX573137
*H. longicornis*	Female	–	China (Sichuan)	China (Sichuan)	JQ346684	JF979373
*H. longicornis*	–	–	–	China (Hebei)	–	JF979374
*H. longicornis*	–	–	–	China (Gansu)	–	FJ712721
*H. qinghaiensis*	–	–	China (Gansu)	China	HQ005302	EF605264
*H. qinghaiensis*	–	Sheep	–	China (Gansu)	–	FJ712720

Note: “−” represent unavailable data. The contents between parentheses represent localities of samples. The accession numbers of our sequences are in bolded characters.

Sequences of ITS2 rDNA and mt 16S were separately aligned using the computer program Clustal X 1.83 [47]. Pairwise comparisons were made of the level of sequence differences (*D*) among the isolates within and among *Haemaphysalis* species using the formula *D* = 1− (*M*/*L*), where *M* is the number of alignment positions at which the two sequences have a base in common, and *L* is the total number of alignment positions over which the two sequences are compared [48], [49].

To infer the taxonomic position of these ticks infesting *A. m. qinlingensis*, phylogenetic relationships of ticks in the present study and *Haemaphysalis* species with available sequences in GenBank™ were re-constructed, using *D. andersoni* (GenBank™ accession numbers S83084 for ITS2 rDNA and EU711328 for mt 16S) as the outgroup (Table 1). All the ambiguously aligned regions were excluded using Gblocks online server (http://molevol.cmima.csic.es/castresana/Gblocks_server.html). The remaining sequences were aligned and used to re-construct the genetic trees using maximum parsimony (MP) method carried out by PAUP 4.0 Beta 10 program [50], in which the bootstrap values were calculated from 1000 bootstrap replicates. Phylograms were drawn using the Tree View program version 1.65 [51].

## Results and Discussion

### Morphological Characterization of Ticks in *A. m. qinlingensis*

Morphological approach is a traditional and authoritative tool to classify taxa of ticks. In last decades, twelve tick species, including three *Ixodes* spp., one *Dermacentor* sp. and eight *Haemaphysalis* spp., were reported in giant pandas [16]–[18]. In the present study, one *Haemaphysalis* species collected from *A. m. qinlingensis* in Qinling Mountains was characterized. Morphological characterization showed similar features with that of *H. flava* Neumann, 1897 [52]. However, limitations of this study were the small number of ticks samples and lack of descriptions of other development stages except adults. The barriers were low number of *A. m. qinlingensis* due to the endanger fact of giant panda [53], [54], and three host ticks of all *Haemaphysalis* spp., for which immature development stages were difficult to obtain [55].

### Molecular Characterization of Ticks in *A. m. qinlingensis*

In some contexts, tick morphological identification is difficult, mainly for immature stages, and ticks collected from wild animals were not identified using molecular evidence. In order to accurately infer the taxa position of *Haemaphysalis* species from *A. m. qinlingensis*, a total of 20 DNA sequences from ten individuals were obtained, including ten ITS2 rDNA sequences and ten mt 16S sequences. After ambiguous bases at both ends discarded, the remaining sequences of ITS2 rDNA and mt 16S were 1164 bp and 367 bp, respectively. The ITS2 rDNA region was high in guanine cytosine (G+C) content with an average of 62.81% (ranged between 62.46% and 62.89%). The sequence variations among ten samples in ITS2 rDNA were 0–0.52%. Pairwise comparison in ITS2 rDNA between ticks from *A. m. qinlingensis* and species in genus *Haemaphysalis* showed that the genetic variations were the lowest between ticks from *A. m. qinlingensis* and *H. flava* (JF758641), with sequence differences of 2.06%–2.40%.

For mt 16S, high nucleotide contents of A+T (77.19%–77.98%) were found for ticks from *A. m. qinlingensis*. The intra-species genetic variations in mt 16S for these ticks were 0–4.09%. Pairwise comparison in mt 16S between ticks from *A. m. qinlingensis* and species in genus *Haemaphysalis* also showed low genetic variations between ticks from *A. m. qinlingensis* and *H. flava* (NC_005292), with sequence variations of 1.30%–4.70%.

**Figure 2:**
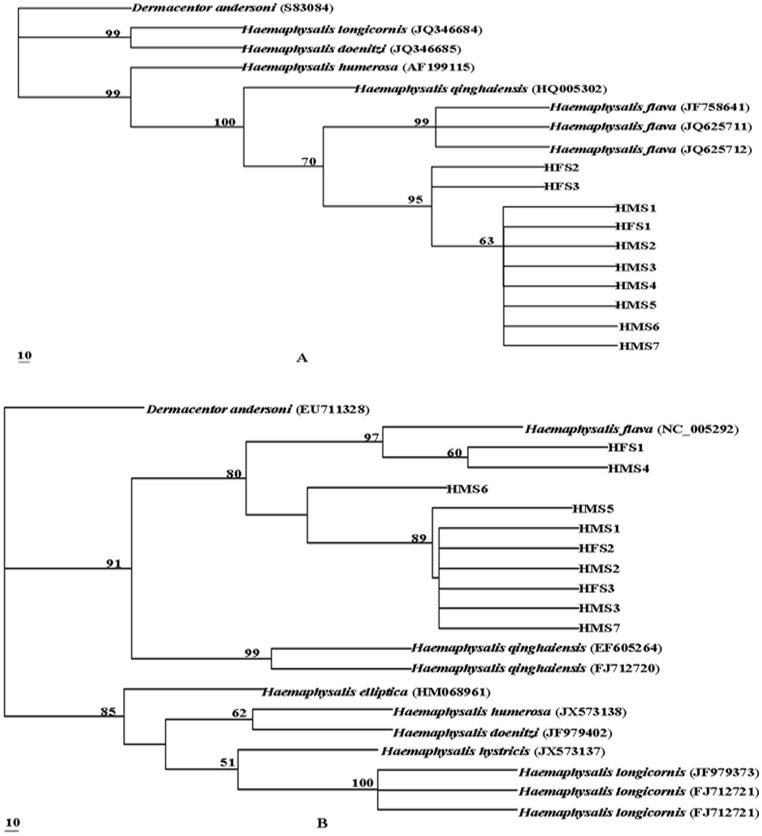
Phylogenetic relationships of ticks isolated from giant pandas with *Haemaphysalis* spp. The *Dermacentor andersoni* was as the outgroup. Phylogenetic analysis was based on ITS2 rDNA (A) and mitochondrial 16S (B) sequences using maximum parsimony (MP) method. The consensus tree was obtained after bootstrap analysis with 1000 bootstrap replicates, with values above 50% reported.

In order to determine the taxonomic status of ticks from *A. m. qinlingensis*, the genetic trees were re-constructed using MP method based on the sequences of ITS2 and mt 16S, respectively. The topological structures of two MP trees were similar. All *Haemaphysalis* species were clustered in two large clades (Figure 2). All ticks from *A. m. qinlingensis* were grouped together sistered to *H. flava* with high bootstrap values (>50%) in ITS2 rDNA tree. Of the mt 16S tree, two samples (HMS1 and HMS4) were located in sister clade of *H. flava* with high bootstrap values (62%), while other tick samples from *A. m. qinlingensis* formed a single clade sistered to clade of *H. flava* and the two samples. These molecular data further confirmed ticks from *A. m. qinlingensis* would be *H. flava*.

The mt 16S and nucleotide ITS2 rDNA have been identified as effective markers for genetic characterization of ticks at inter-species and intra-species levels [22], [26], [46]. However, both of two fragments have some drawbacks in studying population genetics. The ITS2 rDNA tends to be little intraspecific variation but considerable interspecific difference [56]. It is also worth noting that the ITS2 rDNA of *Haemaphysalis* species deposited in GenBank™ varied in length from 1159 bp to 1707 bp. Previous study showed that the ITS2 rDNA of hard ticks apparently evolved mostly by increasing and decreasing lengths of the nucleotide sequences to cause increases and decreases in the length of stems of the secondary structure, and increases in the size of the ITS2 rDNA may have been caused by replication slippage that generated large repeats [29]. Alternatively, the mt 16S seems to be a good marker for the establishment of genetic relationships among closely-related tick species, but it does not seem to be useful for comparisons of distantly related taxa [36], [57]. Therefore, the present study used two fragments to reveal genetic relationships of *Haemaphysalis* species collected from *A. m. qinlingensis* and other *Haemaphysalis* spp. available in GenBank™. Genetic variations between *Haemaphysalis* species collected from *A. m. qinlingensis* and *H. flava* were the lowest in ITS2 rDNA and mt 16S, with 2.06%–2.40% and 1.30%–4.70%, respectively. Phylogenetic relationships showed that the Haemaphysalinae were complicated and polyphyletic, which were consistent with genetic trees re-constructed by Tian et al. [58]. All the *Haemaphysalis* collected from *A. m. qinlingensis* were grouped with *H. flava*, further confirmed that the *Haemaphysalis* sp. in the present study was *H. flava*.

In conclusion, the present study characterized ticks collected from *A. m. qinlingensis* in Qinling Mountains of China. Based on morphological features and molecular evidence, the ticks were identified as *H. flava*. Further study is needed to investigate the prevalence of *H. flava* in sympatric domestic (or wild) animals and whether this tick species transmit any pathogens to animals and human.
